# Nanobody-mediated targeting of Plasmodium falciparum PfPIMMS43 can block malaria transmission in mosquitoes

**DOI:** 10.1038/s42003-025-08033-8

**Published:** 2025-04-30

**Authors:** Chiamaka Valerie Ukegbu, Mgeni Mohamed, Astrid Hoermann, Yuyan Qin, Prisca A. Kweyamba, Dickson Wilson Lwetoijera, Nikolai Windbichler, Sarah Moore, George K. Christophides, Dina Vlachou

**Affiliations:** 1 Department of Life Sciences, Imperial College London, London, UK; 2 Environmental Health and Ecological Sciences, Ifakara Health Institute, Bagamoyo, Tanzania; 3 Swiss Tropical and Public Health Institute, Kreuzstrasse 2, Allschwil, Switzerland; 4 University of Basel, Basel, Switzerland

**Keywords:** Parasitic infection, Pathogens

## Abstract

The transition from ookinete to oocyst is a critical step in the *Plasmodium falciparum* lifecycle and an important target for malaria transmission-blocking strategies. PfPIMMS43, a surface protein of *P. falciparum* ookinetes and sporozoites, is critical for this transition and aids the parasite in evading mosquito immune responses. Previous studies demonstrated that polyclonal PfPIMMS43 antibodies reduced *P. falciparum* infection in *Anopheles* mosquitoes. Here, building on these findings, we have developed high-affinity single-domain VHH antibodies (nanobodies) derived from llama heavy-chain-only antibodies. We have shown that these nanobodies bind both recombinant and endogenous PfPIMMS43 produced by *P. falciparum* ookinetes in the mosquito midgut. Importantly, they significantly reduce infection intensity and prevalence of laboratory and field strains of *P. falciparum* in *An. coluzzii* and *An. gambiae*, respectively. Epitope mapping has revealed that the nanobodies target conserved regions in the second half of PfPIMMS43, with homology modelling confirming epitope accessibility. These findings establish PfPIMMS43 as a promising transmission-blocking target. To enhance malaria control and elimination efforts, we propose an innovative strategy in which genetically modified mosquitoes express PfPIMMS43-specific nanobodies in their midguts and spread this trait in wild mosquito populations via gene drive technology.

## Abstract (web summary)

Nanobodies targeting the ookinete and sporozoite surface protein PIMMS43 block mosquito infection by African *Plasmodium falciparum* field isolates. Gene drive mosquitoes expressing these nanobodies could be deployed to eliminate malaria transmission.

## Introduction

Malaria remains a major global health challenge, with approximately 250 million cases and over 600,000 deaths annually^1^. While significant progress was made in reducing malaria morbidity and mortality in the early 2000s, this trend stalled after 2014 and was further exacerbated by the disruption of public health services due to the COVID-19 pandemic in 2020, leading to a resurgence in cases^2^. To eliminate malaria against a background of climate change, reduced donor funding, failure of insecticidal interventions and massive population growth in malaria endemic areas, it is crucial to develop new interventions that can complement and enhance existing control measures. Ideally, new interventions will be simple to deploy, will not require repeated application and will reduce malaria receptivity of an area for the medium to long term.

Malaria transmission depends on the sexual development of *Plasmodium* in the mosquito vector. When female *Anopheles* mosquitoes ingest male and female gametocytes during a blood meal on a malaria infected human, these gametocytes are activated in the midgut, fuse to form zygotes, and subsequently develop into motile ookinetes. The transition from ookinete to oocyst, occuring as ookinetes traverse the mosquito midgut epithelium within 18–24 h post-blood feeding, is a key developmental bottleneck characterized by significant attrition of ookinetes. This is mediated by the activation of the mosquito JNK (c-Jun N-terminal kinase) signaling pathway, which, through effectors such as heme peroxidase 2 (HPX2) and NADPH oxidase 5 (NOX5), promotes the nitration of ookinetes^3,4^. These ookinetes are then targeted for elimination by the mosquito complement-like system in the hemolymph^5^.

Given the crucial role of human-to-mosquito and mosquito-to-human transmission in sustaining malaria, there is growing interest in developing transmission-blocking interventions that target parasite development within the mosquito. One promising strategy is transmission-blocking vaccines (TBVs), which target parasite stages in the mosquito midgut, including gametocytes, gametes, zygotes and ookinetes, or mosquito midgut antigens, leading to their neutralization or destruction by the human complement system. Leading TBV candidates include the six-cysteine proteins Pfs230 and Pfs48/45 found on gametocyte and gamete surface, as well as the zygote and ookinete surface proteins Pfs25 and Pfs28^6^. Additional candidates include the female gametocyte protein Pfs47, the male gamete fusion protein HAP2, and the mosquito midgut protein APN1.

However, TBV development has been hampered by challenges in producing potent, long-lasting antibodies against key epitopes^7,8^. Advances have been made in identifying conserved protein domains that are easier to express and elicit superior transmission-reduction activity (TRA). For example, Pfs230 domain 1 (D1), now in Phase II clinical trials, and Pfs48/45 C-terminal region (6 C/D3), currently in Phase I trials, have shown promise in inducing transmission-reducing antibodies^9–16^. However, the limited number of high-priority vaccine candidates underscores the need for further antigen discovery. In the post-genomic era, bioinformatic and knockout analyses have identified additional TBV candidates, such as several uncharacterized gametocyte surface proteins, the male gamete proteins PbPH and Pbg37, and ookinete surface proteins PSOP12 and PSOP26, which show promising TRAs^17–21^.

An alternative strategy to TBVs involves genetically modified mosquitoes expressing transmission-blocking antibodies, making them refractory to *Plasmodium* infection. Monoclonal antibodies in the form of single-chain variable fragments (scFv) have shown significant TRA, and transgenic mosquitoes expressing scFvs targeting Pfs25 or chitinase (PfCHT1) have shown reduced parasite loads^22–24^. Recently, smaller, more easily produced monoclonal, heavy-chain variable (VHH) domain antibodies, also known as nanobodies or single-domain antibodies, targeting Pfs230, have demonstrated promising TRA in mosquito assays, with specificity and potency comparable to conventional monoclonal antibodies^25^. These VHH domain nanobodies are fragments of naturally produced single-domain antibodies of camelids, such as alpacas and llamas^26^, and sharks^27^. Due to their small size (~15 kDa), structural simplicity and strong binding affinity^28^, VHH nanobodies are easily bioengineered and are therefore highly attractive for targeting parasite antigens in mosquito vectors.

Previous studies, including ours, have highlighted the potential of polyclonal antibodies targeting *P. falciparum* PIMMS43, also known as PSOP25, in reducing malaria transmission^29–31^. PfPIMMS43 is a GPI-anchored protein expressed on the surface of ookinetes and sporozoites and shown to be indispensable for mosquito midgut traversal by helping parasites to evade the mosquito complement-like system^31^. The aim of this study was to generate and characterize nanobodies targeting PfPIMMS43 and to explore their potential in blocking malaria transmission. Specifically, we sought to generate high affinity nanobodies, map their binding epitopes, and assess their ability to inhibit transmission of both laboratory and field *P. falciparum* in *Anopheles* mosquitoes.

## Results

### Generation and characterization of VHH nanobodies targeting PfPIMMS43

**Fig. 1:**
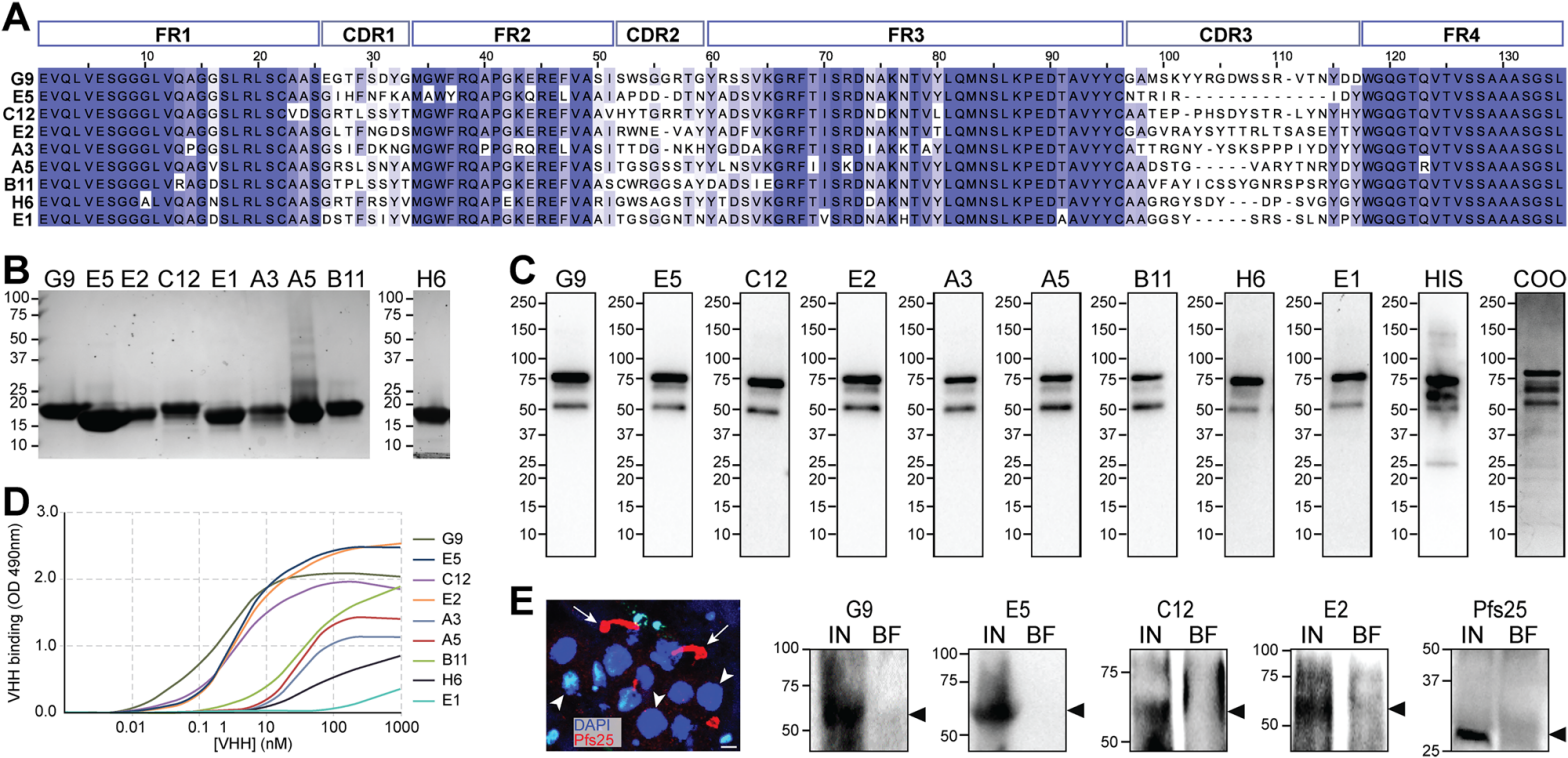
PfPIMMS43 nanobodies and recognition of recombinant and endogenous protein. **A** Multiple sequence alignment of the nine PfPIMMS43-specific nanobodies, highlighting the four framework regions (FR) and three complementarity-determining regions (CDR). Sequence conservation is represented by shades of blue, with complete conservation shown in dark blue and non-conserved regions unshaded. **B** SDS-PAGE gel analysis of recombinant MYC-6xHis-tagged PfPIMMS43 nanobodies expressed in *E. coli* and purified by affinity chromatography. **C** Western blot detection of recombinant thioredoxin-His-tagged PfPIMMS43 using the nine nanobodies. Binding was visualized with an HRP-conjugated anti-VHH antibody. Probing with an anti-His antibody served as a control (HIS). A Coomassie-stained SDS-PAGE gel of recombinant PfPIMMS43 prior to blotting was used as a loading control (COO). **D** ELISA showing nanobody binding affinities to recombinant PfPIMMS43. **E** Detection of endogenous PfPIMMS43 in *P. falciparum* NF54 ookinetes using the four highest affinity nanobodies (G9, E5, C12, and E2). Reduced cell lysates from *P. falciparum*-infected midguts, collected 18 h post-blood meal, were probed with nanobodies, and binding was detected using an anti-MYC antibody. An anti-Pfs25 antibody served as a positive control. The fluorescence microscopy image shows ookinetes (red, stained with anti-Pfs25) invading midgut epithelial cells (nuclei stained blue with DAPI), corresponding to midgut samples used for western blot analysis. IN, infected midguts; BF, blood-fed midguts. Arrows in the immunofluorescence image indicate Pfs25-stained ookinetes and arrowheads indicate DAPI-stained epithelial cell nuclei. Smaller nuclei correspond to other cells in the midgut epithelium. Scale bar, 5 µM.

PfPIMMS43 is a 505-amino acid protein with an N-terminal signal peptide and a C-terminal GPI anchor. To generate nanobodies against PfPIMMS43, we produced a recombinant Thioredoxin-His-tagged version of PfPIMMS43, excluding the signal peptide and GPI anchor sequences (amino acids 26-481), in *Escherichia coli* as previously described (Ukegbu et al., 2020). This recombinant protein was used to immunize llamas, generating an immune nanobody library from which nine nanobodies (G9, E5, C12, E2, A3, A5, B11, H6, and E1) were selected. These nanobodies were chosen based on variations in their antigen-binding complementary determining regions (CDR1-3) (Fig. 1A). CDR1 and CDR2 showed median sequence identities of 25% and 22%, respectively, while CDR3, the region most involved in antigen binding, displayed a median sequence identity of less than 18% between the nine nanobodies. The length of CDR1 and CDR2 was consistently eight amino acids, except for the 7-amino acid CDR2 found in E5, E2, and A3 nanobodies. The CDR3 length varied significantly, ranging from 8 to 21 amino acids (median 15), with E5 having the shortest CDR3 (Fig. 1A). Despite the CDR diversity, the framework regions (FRs) were highly conserved, with some variations in the first and middle regions of FR3. Nanobodies are known to sometimes utilize FR regions to enhance antigen-binding diversity^32^.

All nine nanobodies were expressed in *E. coli* as Myc-His fusion proteins, which under non-reducing conditions on SDS-PAGE gels migrated between 15 and 20 kDa (Fig. 1B). To assess the ability of nanobodies to recognize recombinant TRX-His-PfPIMMS43, we performed western blot analyses using an anti-VHH antibody. All nine nanobodies successfully recognized the full-length (FL) recombinant PfPIMMS43 (~75 kDa) under non-reducing conditions (Fig. 1C, Fig. S1). Two lower bands detected were likely degradation products of recombinant PfPIMMS43, as they were also detected by the anti-His antibody.

The binding affinities of the nanobodies were evaluated using ELISA (Fig. 1D). Four of the nanobodies, G9, E5, C12, and E2, exhibited high nanomolar binding affinities to recombinant PfPIMMS43 (3, 5, 6, and 8 nM, respectively). The remaining 5 nanobodies demonstrated lower binding affinities, greater than 50 nM.

Therefore, we selected the four nanobodies with the highest affinity (G9, E5, C12, and E2) and investigated their ability to detect the ~60 kDa PfPIMMS43 protein expressed in *P. falciparum* ookinetes. Previous studies using polyclonal antibodies demonstrated that PfPIMMS43 is expressed on the surface of invading ookinetes 18–25 h post blood feeding (Ukegbu et al., 2020). Western blot analyses of *P. falciparum* NF54-infected *An. coluzzii* midguts at 18 h post blood feeding revealed that all four nanobodies detected a ~ 60 kDa band corresponding to PfPIMMS43 (Fig. 1E, Fig. S2). This band was absent in non-infected blood-fed midguts, which served as controls. As a stage-specific control for PfNF54 ookinete presence, Pfs25 was detected in infected midguts but not in the blood-fed controls.

### Transmission blocking of *P. falciparum* by PfPIMMS43 nanobodies

**Fig. 2:**
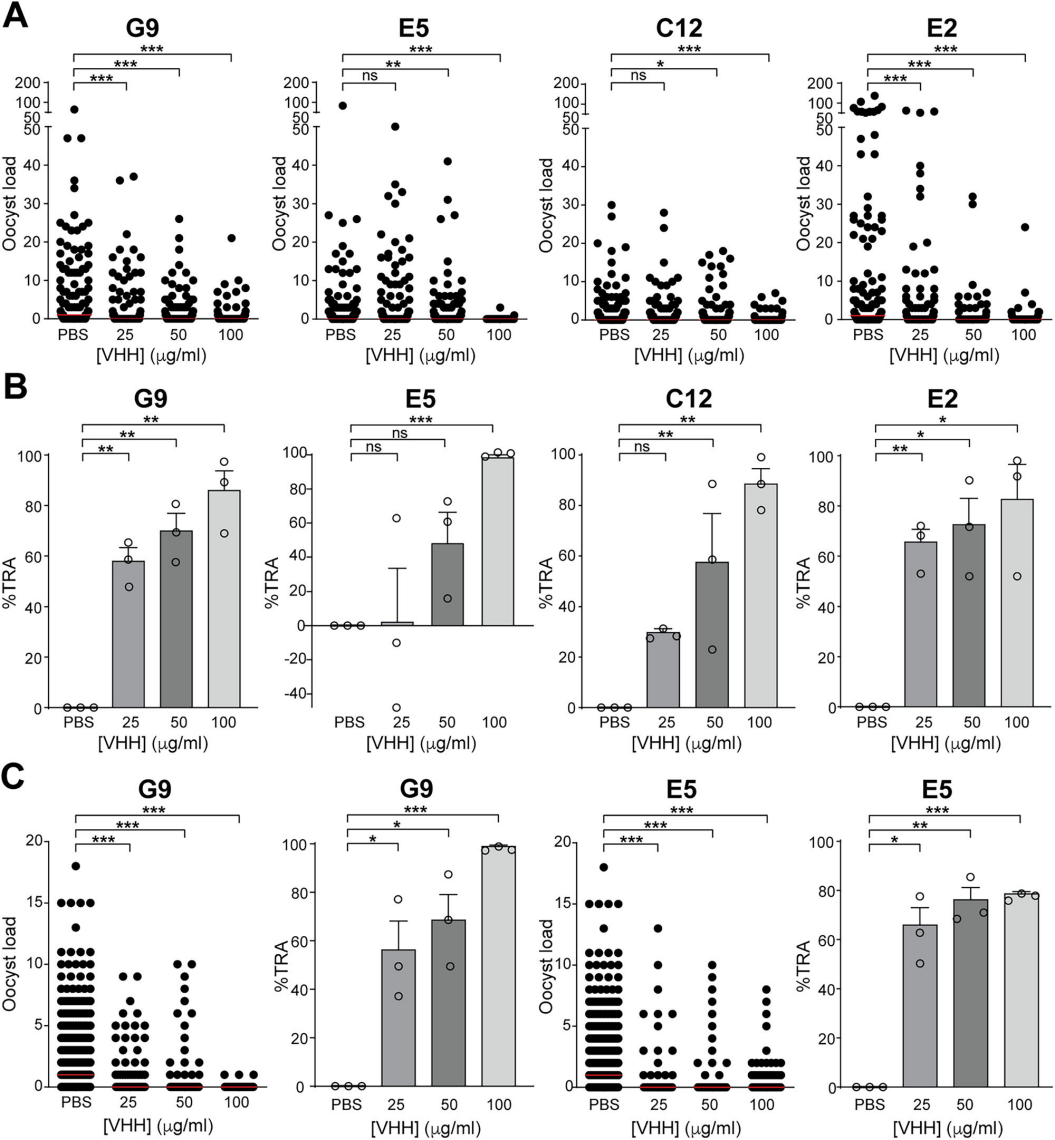
PfPIMMS43 nanobody performance in transmission assays. **A** Oocyst counts per midgut at 8–10 days post-blood meal from SMFAs using *An. coluzzii* N’gousso mosquitoes infected with *P. falciparum* NF54 gametocytes. Gametocytes were treated with G9, E5, C12, and E2 nanobodies, with PBS-treated gametocytes serving as the control. Data from three biological replicates per nanobody treatment were pooled. Red horizontal lines indicate the median oocyst count. Statistical significance was assessed using the Mann–Whitney test: ns, not significant; *P < 0.05; **P < 0.001; ***P < 0.0001. **B** Percent transmission-reducing activity (TRA) of the PfPIMMS43 nanobodies G9, E5, C12, and E2 relative to the PBS control, calculated from the results in **A**. Circles represent individual data. Error bars represent the SEM. Statistical significance was determined using an unpaired t-test with Welch’s correction: ns, not significant; *P < 0.05; **P < 0.001; ***P < 0.0001. **C** Oocyst counts per midgut at 8–10 days post-blood meal from DMFAs using *An. gambiae* Ifakara mosquitoes infected with *P. falciparum* gametocytes collected from children in Tanzania. Gametocytes were treated with G9 and E5 nanobodies, with PBS-treated gametocytes as the control. Data from three biological replicates were pooled. Statistical analyses of oocyst load and TRA were performed as in **A**, **B**.

We assessed the ability of the G9, E5, C12, and E2 PfPIMMS43 nanobodies to block *P. falciparum* NF54 transmission using *An. coluzzii* standard membrane feeding assays (SMFAs). Oocyst numbers in mosquito midguts were counted 8–10 days post-infectious blood feeding. Phosphate-buffered saline (PBS) was used as the control, as nanobodies were dissolved in this solution. No significant difference in oocyst intensity was observed when *P. falciparum* NF54 gametocytes were fed to *An. coluzzii* compared to those spiked with PBS (Table S1). We tested three different nanobody concentrations, 25, 50, and 100 µg/ml, and found that oocyst reduction was concentration-dependent, with the greatest inhibition observed at the highest concentration (Fig. 2A, B, Table S1). At the highest concentration of 100 µg/ml, G9, E5, C12, and E2 significantly reduced oocyst numbers by 86%, 99%, 89%, and 83%, respectively, compared to the PBS control (Fig. 2B, Table S1). For G9 and E2, significant TRA were also observed at both 50 µg/ml and the lowest concentration of 25 µg/ml: 70% and 58% for G9, and 73% and 66% for E2, respectively. C12 showed significant 58% TRA at 50 µg/ml but not at 25 µg/ml concentrations, while E5 showed significant 99% TRA at a 100 µg/ml concentration, the highest of all nanobodies (Fig. 2B, Table S1).

Next, we tested the ability of G9 and E5, the two nanobodies with the highest affinities to recombinant PfPIMMS43, to block the transmission of natural *P. falciparum* isolates. Using direct membrane feeding assays (DMFAs) on blood samples from gametocytaemic children in Tanzania (Table S2), spiked with G9 and E5 nanobodies at 25, 50, and 100 µg/ml, we counted oocyst numbers 8–10 days post blood feeding of local *A. gambiae* mosquitoes (Ifakara strain). *A. coluzzii* is not endemic in Tanzania, and the two species are very closely related and often used interchangeably in both laboratory and field settings. The results were consistent with the laboratory-based SMFAs, with the highest TRA observed at 100 µg/ml for both nanobodies (Fig. 2C, Table S3). At this concentration, G9 and E5 reduced oocyst numbers by 99% and 79%, respectively, from an average of 2.3 to 0 and 0.5 oocysts per midgut, respectively (Fig. 2C, Table S3). G9 also significantly reduced transmission by 56% at 25 µg/ml and 68% at 50 µg/ml. Interestingly, E5 performed better against field *P. falciparum* isolates than against the laboratory NF54 strain, significantly reducing infection by 66% at 25 µg/ml and 76% at 50 µg/ml. Both nanobodies significantly reduced the mosquito infection prevalence in all concentrations tested (Table S3). Raw data obtained from infections with laboratory NF54 and natural *P. falciparum* isolates are presented in Supplementary Data File 1.

### Epitope mapping of VHH-PfPIMMS43 interactions

**Fig. 3:**
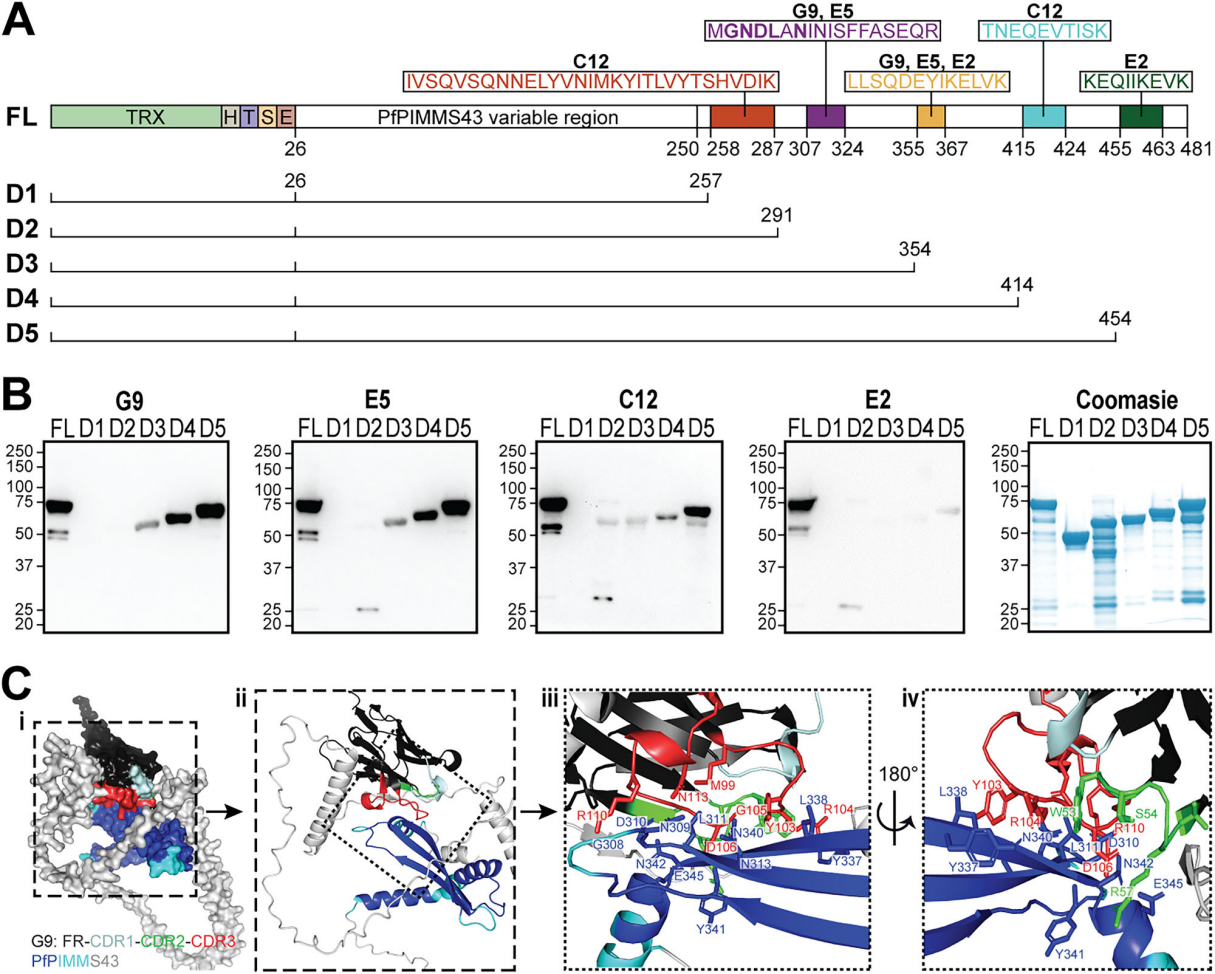
PfPIMMS43 epitope mapping of VHHs and 3D homology modeling. **A** Schematic representation of the full-length (FL) recombinant PfPIMMS43 protein and its subdomains D1-D5, created through progressive C-terminal deletions. Amino acid sequences corresponding to the regions bound by each tested nanobody, as determined by LC-MS/MS following VHH-PfPIMMS43 binding and trypsin digestion, are shown. All constructs were expressed in *E. coli* as Thioredoxin-His fusion proteins. TRX-Thioredoxin, H-6xHis, T-Thrombin cleavage site, S-S-tag and E- enterokinase cleavage site. **B** Western blot detection of recombinant Thioredoxin-His-tagged PfPIMMS43 FL and its truncated variants D1-D5 using G9, E5, C12, and E2 nanobodies. Each nanobody was tested individually, and binding was detected using an HRP-conjugated anti-VHH antibody. A Coomassie-stained SDS-PAGE gel of the recombinant PfPIMMS43 proteins, served as a loading control. **C** Homology model of the G9-PfPIMMS43 complex. PfPIMMS43 is colored based on pLDDT confidence scores: pLDDT > 90 in blue, 90 > pLDDT > 70 in cyan, and pLDDT<70 in fading shades of silver. G9 is represented with framework regions (FR) in black, CDR1 in pale cyan, CDR2 in green, and CDR3 in red. Panels: (**i**) surface representation of the G9-PfPIMMS43 complex; (**ii**) cartoon representation of the region indicated in panel (i); (**iii**) zoom-in of the region indicated in the panel (ii) with side chains of interacting residues from G9 CDR2 (green), CDR3 (red) and PfPIMMS43 (blue) shown; (**iv**) Back side of the region shown in panel (iii) with side chains of interacting residues shown.

The epitopes of the PfPIMMS43 protein recognized by the G9, E5, C12, and E2 nanobodies were identified using an antigen-binding and enzyme-digestion assay. Immobilized nanobodies were bound to recombinant TRX-His-PfPIMMS43, and the resulting VHH-PfPIMMS43 complexes were digested with trypsin. Eluted peptides from the bound complex were then compared to non-bound peptides and a negative control. Several unique peptides, present only in the nanobody-bound fraction, were identified (Supplementary Data File 2), and mapped to the PfPIMMS43 sequence (Fig. 3A). Some of these epitopes were bound by more than one of the nanobodies but some were unique for different nanobodies.

To validate the importance of these identified peptides for nanobody recognition, subdomains of PIMMS43 spanning different epitopes were designed, expressed and tested for binding with the nanobodies. Like the full-length PfPIMMS43 spanning amino acids 26-481 (~74 kDa), lacking the N-terminal signal peptide and C-terminal GPI-anchor sequence, these subdomains were produced as thioredoxin-His-tagged fusion proteins (Fig. 3A). They included: Domain 1 (D1) spanning amino acids 26-257 (47 kDa), D2 spanning amino acids 26-291 (51 kDa), D3 spanning amino acids 26-354 (58 kDa), D4 spanning amino acids 26-414 (65 kDa), and D5 spanning amino acids D26-K454 (69 kDa).

In western blots, compared to the full-length PIMMS43, G9 did not bind to subdomains D1 and D2 but was able to bind to D3, D4, and D5 (Fig. 3B). The binding was associated with two putative peptide epitopes: MGNDLANINISFFASEQR, found in D3, D4, and D5, and LLSQDEYIKELVK, present only in D4 and D5. E5 displayed a similar binding profile to G9, suggesting that it recognizes the same epitope bin (Fig. 3B). For C12, the strongest binding was observed with subdomain D5, which contains the putative epitope TNEQEVTISK, suggesting that this specific region is critical for C12 recognition (Fig. 3B, Fig. S3). Finally, while E2 showed some recognition of subdomains D4 and D5, which contains the epitope LLSQDEYIKELVK, the relatively weak signal to D4 suggests that the C-terminal peptide epitope KEQIIKEVK in D5 may be essential for recognition by E2 (Fig. 3B).

### Homology modeling of VHH-PfPIMMS43 interactions

We used AlphaFold to generate a 3D homology model of the G9 nanobody binding to PfPIMMS43 (Fig. 3C). Initial attempts to model the interaction with the full-length PfPIMMS43 could not produce a confident 3D structure, as the protein contains large unstructured regions. Indeed, earlier attempts to analyze the structure of recombinant PfPIMMS43 using nuclear magnetic resonance (NMR) suggested that much of the protein is unfolded. Subsequently, the model was refined to focus on the most confidently modeled PfPIMMS43 region, spanning amino acids 44-367 and encompassing three of the predicted epitopes determined by the mapping assay (Fig. 3A). The resulting model of the PfPIMMS43-G9 complex had an inter-residue predicted TM-score (ipTM) of 0.81 and a predicted TM-score (pTM) of 0.51, indicating a reasonable level of confidence.

The folding of PfPIMMS43 was predicted with high confidence (pLDDT > 90) for amino acid residues 250–352, particularly for residues 292–301, 311–323, and 331–342, which together form a β-sheet structure (Fig. 3Ci). Three α-helices (amino acids 250–263, 266–282, and 344–352) showed moderate confidence (pLDDT > 70). The G9 nanobody structure displayed the classical β-sandwich immunoglobulin fold, with high confidence (pLDDT > 90) in the fold, and moderate confidence (90 > pLDDT > 70) for the CDR loops (Fig. 3Ci).

Both cartoon and surface representations of the model indicated that G9 binds to PfPIMMS43 primarily through its CDR2 and CDR3 domains, with the interaction dominated by CDR3 (Fig. 3Cii, iii). A detailed analysis of sidechain residues revealed that CDR3 interacts with residues in the high-confidence β-sheet of PfPIMMS43, including G308, N309, D310, L311, N313, Y337, L338, N340, and N342. CDR2 engages residues Y341 and N342 from the β-sheet, as well as E345 from an adjacent α-helix (Fig. 3Civ). The predicted aligned error (PAE) for these interactions was less than 5 Å, further supporting the accuracy of the predictions. Importantly, residues G308, N309, D310, L311, and N313 are part of the predicted peptide epitope MGNDLANINISFFASEQR (Fig. 3A). Attempts to model interactions of E5, C12, and E2 with PfPIMMS43 were unsuccessful.

## Discussion

We report the characterization of VHH domain nanobodies, derived from llama heavy-chain-only antibodies, targeting PfPIMMS43, a crucial protein for the malaria parasite’s transition from ookinete to oocyst and its evasion of the mosquito immune system. Four nanobodies, G9, E5, C12, and E2, demonstrated significant TRA in laboratory infections of *An. coluzzii* with *P. falciparum* NF54, reducing both the intensity and prevalence of oocysts in mosquito midguts. Notably, two of these nanobodies, G9 and E5, also replicated this effect in DMFAs with *An. gambiae* using natural *P. falciparum* gametocyte isolates from malaria infected children in sub-Saharan Africa.

Previous studies have shown that monoclonal and polyclonal antibodies against rodent *P. berghei* PIMMS43 exhibit significant TRA^29–31^. More recently, a nanoparticle-based vaccine targeting PbPIMMS43 demonstrated moderate TRA^33^. Additionally, polyclonal antibodies against the human orthologs PvPIMMS43 and PfPIMMS43 have been shown to confer TRA significant against *P. vivax* clinical isolates^34^ and laboratory-cultured *P. falciparum* NF54, respectively^31^. Together with the findings reported here, these data position *PfPIMMS43* as a critical target for malaria transmission-blocking strategies.

The putative epitopes for the four nanobodies showing the highest affinity for *P. falciparum* NF54 PIMMS43 were determined. Notably, all four nanobodies bound to epitopes located in the highly conserved and structured second half of the protein beyond amino acid residue 258. This finding suggests that the variable N-terminal half of PfPIMMS43 may be less immunogenic compared to the C-terminal half of the protein. Further investigation is needed to determine whether this N-terminal region, which is absent in rodent PIMMS43 orthologs and present only in *P. falciparum* and *P. vivax* PIMMS43^31^, can elicit potent transmission-reducing antibodies or nanobodies. G9 and E5 nanobodies appear to recognize the same conformational epitope despite significant amino acid sequence differences in their CDR regions; notably, the CDR3 of E5 contains only eight amino acids compared to 20 amino acids in G9. Unfortunately, we could not generate an accurate homology model of the E5 interaction with PIMMS43, preventing a direct comparison with the model of PfPIMMS43 interaction with G9. These observed differences in the two nanobodies may result in distinct binding patterns and kinetics to the same epitope, potentially explaining the varying potencies observed in the SMFAs.

The epitopes for C12 and E2 nanobodies appear to be linear and different from each other and from those of G9 and E5, which interact with the more structured region of PIMMS43. Both linear epitopes are positioned closer to the C-terminus of PIMMS43, which is thought to be GPI-anchored to the parasite membrane, thus making it less exposed on the ookinete surface compared to the G9 and E5 epitopes. However, the location of these epitopes does not seem to affect the potency of C12 and E2 nanobodies in SMFAs, suggesting that they remain easily accessible on the parasite surface.

Homology modeling of the G9 nanobody bound to PfPIMMS43 using AlphaFold was limited by the fact that high-confidence predictions were only obtained for the PfPIMMS43 region encompassing amino acids 250–352. The lack of significant homology between PfPIMMS43 and known proteins likely impeded AlphaFold’s performance. Preliminary structural analysis suggests that PfPIMMS43 is largely intrinsically disordered, which aligns with the data from AlphaFold modeling and further supports our previous hypothesis that PfPIMMS43 plays a role in immune evasion (Ukegbu et al. 2020). This flexibility, characteristic of disordered proteins, may facilitate the “masking” of the ookinete, shielding it from the mosquito complement-like system. Similar immune evasion strategies, termed “conformational masking,” have been observed in the HIV-1 envelope glycoprotein^35^. The homology model suggested that the G9 nanobody accesses its conformational surface-exposed epitope via its CDR3 from the top of PfPIMMS43. However, the full interaction of G9 with its epitope could not be modeled. Future studies employing techniques such as circular dichroism (CD) spectroscopy and crystallography will be crucial for determining the precise secondary structure of PfPIMMS43 and elucidating the complete binding mechanisms of G9 and the other three nanobodies.

PfPIMMS43 is a post-fertilization target, similar to other TBV candidates like Pfs25, which are expressed only during parasite stages within the mosquito midgut. Although vaccination-induced antibodies can potentially neutralize *Plasmodium* at these stages, the example of Pfs25 highlights a significant limitation: clinical trials have consistently struggled to achieve antibody titres high enough to provide potent TRA^36–38^. A primary challenge for TBVs targeting post-fertilization proteins such as Pfs25 is the absence of natural immune boosting, as these proteins are not expressed in humans. However, PfPIMMS43 holds unique potential, as it is re-expressed in sporozoites^31^. While its role at this stage remains uncharacterized, PfPIMMS43 could therefore be a target of natural antibodies against pre-erythrocytic stages, presenting an opportunity for natural immune boosting. Whether these antibodies exist in malaria-infected individuals is a question that warrants further exploration.

To overcome the challenge of low and unsustainable antibody titers against mosquito-stage parasite antigens, we propose a novel approach: genetically modifying mosquitoes to express nanobodies targeting key post-fertilization proteins such as PfPIMMS43, with the possibility of spreading these traits in wild populations via gene drive technologies. Population genetic studies of *P. falciparum* isolates from Africa reveal that PfPIMMS43 is largely conserved across regions^31^. However, single nucleotide polymorphisms (M307I, L311I, L355H, L355P) with high fixation indices and signatures of positive selection have been identified in the epitope recognized by the G9 and E5 nanobodies, suggesting possible evolutionary adaptations of the sporozoites to evade immune detection in the human host. The hypothesis that these alleles represent an immune evasion mechanism, along with their impact on nanobody recognition of ookinete PfPIMMS43, requires further investigation to understand potential implications for field applications.

## Materials and methods

### Generation of nanobodies against PfPIMMS43

Recombinant PfPIMMS43^31^, comprising the Pfc43opt fragment (amino acids 25–481) without the signal peptide and C-terminal hydrophobic domain and carrying N-terminal TRX and 6xHis tags, along with a C-terminal 6xHis tag, was expressed and purified as previously described^31^.

Nanobodies against recombinant PfPIMMS43 were generated by Eurogentec. Two llamas were immunized with recombinant PfPIMMS43, and lymphocyte RT-PCR was performed to obtain cDNA libraries. These libraries were cloned into the pQ81 phagemid vector and electroporated into *E. coli* TG1 cells for recombinant phage expression, resulting in immune nanobody libraries with a size of more than 10^8^ clones per library per animal and 100% correct insert frequency. Phage selection underwent two rounds, with counterselection against TRX. From 78 sequenced clones, nine nanobodies were selected based on binding results from peri-ELISA assays and amino acid differences in CDR1-3 regions.

### Purified VHH binding affinity by ELISA

VHH affinity was determined using dose response ELISA. Briefly, MaxiSorp plates were coated with 2 μg/mL (50 uL) recombinant PfPIMMS43 in PBS and incubated overnight. The PIMMS43 coated plates were washed with PBS and then blocked with 4% (w/v) skimmed milk powder in PBS. Next, the plate wells were incubated with VHH, with a starting concentration of 1000 nM and then serially diluted 1:10 for 1 h at room temperature. Bound VHH are detected with rabbit-anti-VHH, followed by donkey-anti-rabbit HRP. In between incubations, plates are washed with PBS with 0.05% (v/v) Tween-20 or PBS. Bound HRP-coupled VHH was detected using o-phenylenediamine dihydrochloride (OPD) with H_2_O_2_ as substrate with converted substrate measured at 490 nm wavelength.

### Nanobody expression and purification

PfPIMMS43 nanobodies were expressed in *E. coli* BL21 (DE3) cells. Cultures were grown at 37 °C in 2 x YT media supplemented with 100 µg/mL ampicillin and 0.1% (w/v) glucose until the OD_600_ reached 0.6. Protein expression was induced with 1 mM IPTG, and cultures were further grown overnight at 16 °C. Cells were harvested and lysed using Cell-Lytic (Merck) containing protease inhibitors (cOmplete EDTA-free, Roche). After centrifugation to remove debris, nanobodies were purified as Myc-His-fusion proteins via cobalt affinity chromatography using TALON® metal affinity resin under native conditions in PBS, pH 7.4. The affinity-purified nanobodies were further subjected to size exclusion chromatography for final purification.

### Laboratory *P. falciparum* maintenance, gametocyte culture, and mosquito infection

*P. falciparum* NF54 was cultured as described previously^39^. Asexual cultures were maintained in 10 mL complete medium (RPMI-1640-R5886 supplemented with 0.05 g/L hypoxanthine, 0.3 mg/L L-glutamine, and 10% (v/v) sterile human A+ serum from batch-tested sources for gametocyte production) containing human A+ red blood cells at 5% hematocrit. Cultures were diluted weekly to maintain parasitemia below 10%, and media were refreshed daily. Cultures were gassed with a 5% O_2_, 5% CO_2_, and 90% N_2_ mixture and incubated at 37 °C. Gametocyte cultures were initiated by diluting asexual cultures to 1% ring forms in 8 mL reduced complete medium at 5% hematocrit and maintained for 14 days with daily gas and media changes. *An. coluzzii* mosquitoes, maintained at 27 °C with 70–80% relative humidity and a 12:12 h light-dark cycle, were infected via SMFAs. Briefly equal volume of gametocytes was added to 1.5 mL tubes Eppendorf tubes containing the nanobodies at different concentrations, mixed and then transferred to glass feeders for mosquito infection. Mosquito midguts were dissected 8–10 days post blood feeding, stained with 2% mercurochrome and assessed for infection under light microscopy.

### Field *P. falciparum* mosquito infection

Field studies were conducted as previously described^40^. Briefly, blood samples were collected by finger prick from asymptomatic children aged 6-15 in primary schools. Thick blood smears from those who were tested positive with rapid diagnostic test (RDT) were stained with 10% Giemsa and analyzed microscopically for *P. falciparum* asexual and gametocyte stages. Gametocyte and asexual stages were counted in observed fields that cumulatively contained at least 500 and 200 white blood cells (WBC) respectively and parasite densities were calculated assuming 8000 WBC per µL of blood. Assenting asymptomatic gametocyte carriers were enrolled with parental consent, while children with mixed *Plasmodium* infections were excluded. Venous blood was collected from participants using heparinized vacutainers. The samples were then centrifuged for 3 min at 2000 rpm after which the serum was replaced with equal volume of non-immune AB+ serum. Each feeder was supplied with 300 µl of infectious blood spiked with the nanobodies at varying concentrations. For the control group, 300 µl of blood without nanobodies was used. *An. gambiae sensu stricto* (Ifakara strain) mosquitoes, maintained at 27 ± 2 °C with a relative humidity of 70–80% and 12–12 h of dark-light cycle, were infected through DMFAs. The mosquitoes were allowed to feed for 15 min. Unfed mosquitoes were removed using a mouth aspirator, while the remaining mosquitoes were provided with a 10% sugar solution. The cups containing the mosquitoes were then placed in plastic cages (30 × 30 × 30 cm) inside a climatic chamber (AraLab, Portugal) set to 75 ± 2% humidity and 27 ± 1 °C, following a 12:12 h dark:light cycle. Mosquito midguts were dissected 8–10 days post blood feeding, stained with 2% mercurochrome and assessed for infection under light microscopy. All children found to be malaria positive were treated within 24 hours after detection at the local clinic.

### Western blotting

Midguts were lysed in cell lysis buffer (1x PBS, 1% v/v Triton X-100) containing protease inhibitors. Lysates were boiled under reducing conditions in Laemmli buffer and separated on 10% SDS-PAGE gels. Proteins were transferred to PVDF membranes (GE Healthcare) and detected using primary antibodies: rabbit α-myc (CST, 1:500), rabbit α-VHH-HRP (Genscript, 1:1000), and 4D7 mouse monoclonal α-Pfs25 (1:100). Secondary HRP-conjugated goat α-rabbit IgG and goat α-mouse IgG antibodies (Promega) were used at 1:5000 dilution. All antibodies were diluted in 5% milk-PBS-Tween (0.1% v/v).

### Nanobody epitope mapping

NHS-activated magnetic beads (Pierce™ NHS-Activated Magnetic Beads) were prepared as per manufacturer instructions. Briefly, 300 µL of beads was washed with 1 mM HCl and incubated overnight with 150 µg of VHH nanobodies in 300 µL coupling buffer (50 mM borate, pH 8.5) at 4 °C. For the negative control, 300 µL of washed beads were also incubated with 300 µL of 1 mg/mL PfPIMMS43 in coupling buffer. Unreacted sites were blocked with 0.1 M glycine, pH 9, and the reaction quenched with 3 M ethanolamine, pH 9.0. The beads were stored in coupling buffer until use.

For PfPIMMS43 binding, 300 µL of 1 mg/mL PfPIMMS43 in coupling buffer was added to immobilized VHH beads and incubated at room temperature for 2 h with slow rotation. Beads were washed three times with 50 mM NH_4_HCO_3_, pH 7.8, to remove unbound proteins. Trypsin (5 µL of 1 mg/mL) was added for proteolytic degradation in a final volume of 100 µL NH_4_HCO_3_ buffer and incubated at 37 °C for 4 h. Supernatants were collected, and the beads washed with 1xPBS buffer to remove unbound cleavage products. Bound peptides were eluted with 0.1 M glycine, pH 2.0, and the supernatants collected for mass spectrometry.

PfPIMMS43 subdomains were generated by progressive C-terminal deletions. This was carried out by mutating in the original pET-32b vector containing the *E. coli* codon-optimized PfPIMMS43 fragment (amino acids 25–481) (see above), aa residues 258, 292, 355, 415, 455 to the stop codon (TGA) using Quickchange site directed mutagenesis kit (Agilent) to produce PfPIMMS43 D1, D2, D3, D4 and D5 respectively. Recombinant PfPIMMS43 D1-D5 proteins were expressed and purified as the original PfPIMMS43 (see above).

### Nanobody 3D homology modeling

Nanobody tertiary structures were predicted using AlphaFold, and 3D structures were visualized and colored in PyMOL.

### Statistics

For statistical analysis of *Plasmodium falciparum* mosquito infection intensity, P-values from three biological replicates were calculated using the Mann–Whitney U-test. Statistical analysis for *P. falciparum* transmission reduction was performed using a two tailed, unpaired Student’s t-test with Welch’s correction. All analyses were performed using GraphPad Prism v8.0

### Inclusion and Ethics

This study was conducted with a strong commitment to inclusion, ethics, and local engagement. We adhered to rigorous ethical standards and collaborated closely with local communities, ensuring that all stakeholders were respected. Local researchers were integral to every phase of the research, from study design to data management and co-authorship of publications. The research was tailored to the local context, in partnership with the Ifakara Health Institute, with clear roles and responsibilities outlined from the outset. We designed the study to minimize any risks of harm, discrimination, or stigmatization to participants. No biological materials, cultural artifacts, or traditional knowledge were exported from the country. The study protocol was reviewed and approved by the IHI Institutional Biosafety Committee (IBC) and the National Institute for Medical Research, Tanzania (Ethical Clearance No. NIMR/HQ/R.8c VOL.I/2545). Written informed consent was obtained from parents or guardians, and oral assent was provided by the participating children. All research procedures adhered to ethical guidelines, including the Declaration of Helsinki, ensuring that participants’ rights to withdraw from the study and their privacy were fully respected throughout.

### Reporting summary

Further information on research design is available in the Nature Portfolio Reporting Summary linked to this article.

## Supplementary information

Supplementary Material

Description of Additional Supplementary Materials

Supplementary Data 1

Supplementary Data 2

Reporting Summary

## Data Availability

All data supporting the findings of this study are available within the paper and its Supplementary Information. Numerical source data for Fig. 2 was provided in Supplementary Data File1. Raw data of Fig. 3 was provided in Supplementary Data File 2. Uncropped and unedited blot images for the Fig. 1, Fig. 2 and Fig. 3 are presented in Supplementary Fig. S1, S2 and S3 respectively.
