# Supplementary material for: Nanobody-mediated targeting of Plasmodium falciparum PfPIMMS43 can block malaria transmission in mosquitoes
Source: Commun Biol. 2025 Apr 30;8:683. doi: 10.1038/s42003-025-08033-8 (PMC12041390; doi:10.1038/s42003-025-08033-8)
Supplement: Supplementary file 1 — Supplementary Material [file 42003_2025_8033_MOESM1_ESM.pdf]

Nanobody-mediated targeting of *Plasmodium falciparum* PfPIMMS43 can block malaria transmission in mosquitoes

Chiamaka Valerie Ukegbu[1], Mgeni Mohamed[2], Astrid Hoermann[1], Yuyan Qin[1], Prisca A Kweyamba[2], Dickson Wilson Lwetoijera[2], Nikolai Windbichler[1], Sarah Moore[2,3,4], George K. Christophides[1], Dina Vlachou[1]*

[1]Department of Life Sciences, Imperial College London, London, SW7 2AZ, United Kingdom

[2]Environmental Health and Ecological Sciences, Ifakara Health Institute, Bagamoyo, P.O. Box 74, Tanzania

[3]Swiss Tropical and Public Health Institute, Kreuzstrasse 2, 4123 Allschwil, Switzerland

[4]University of Basel, Petersplatz 1, 4001 Basel, Switzerland

*Correspondence and requests of materials should be addressed to Dina Vlachou, email: d.vlachou@imperial.ac.uk

Supplementary Figures

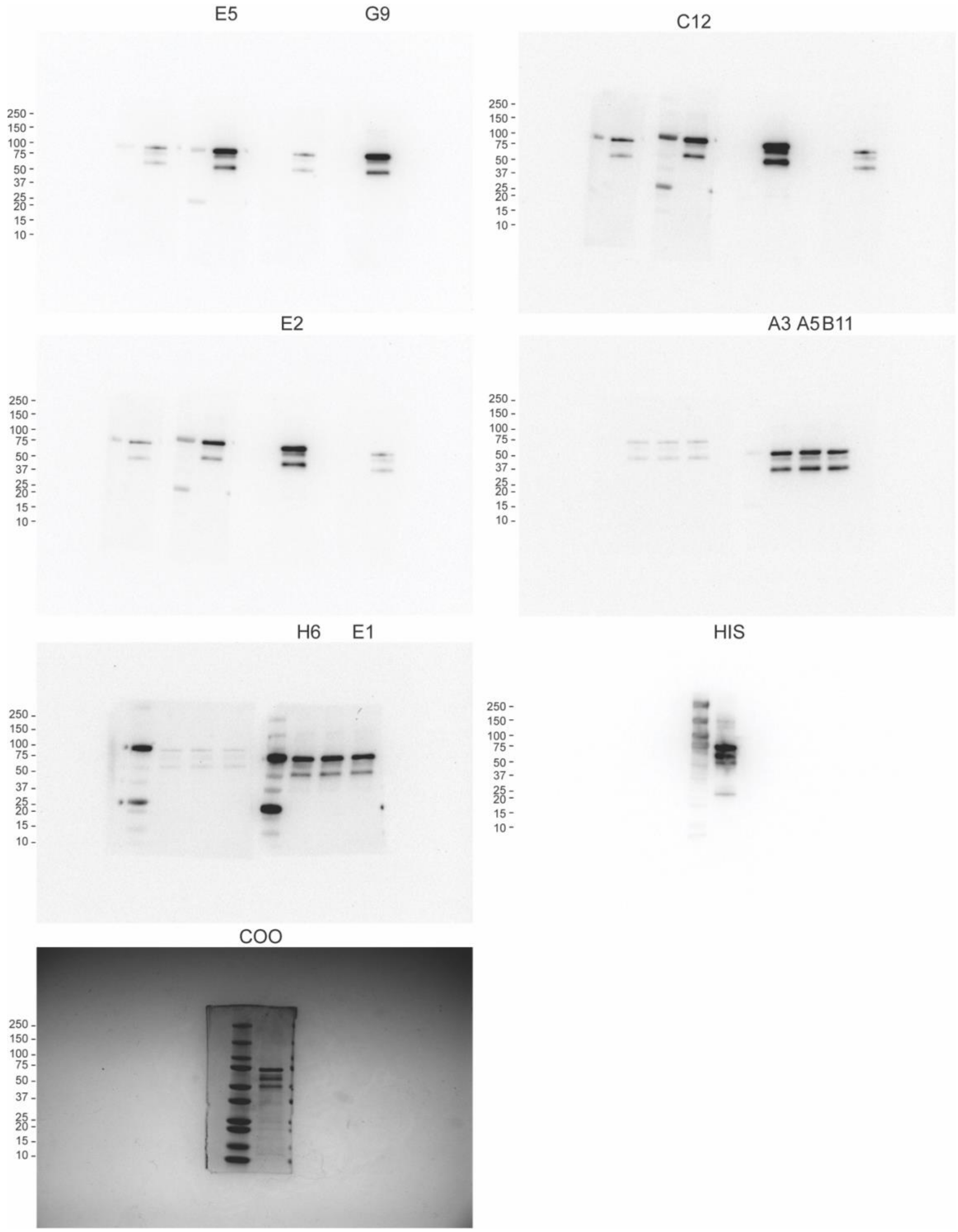

Figure S1. Nanobody recognition of recombinant PfPIMMS43 protein.
Western blot detection of recombinant thioredoxin-His-tagged PfPIMMS43 using the nine nanobodies. Binding was visualized with an HRP-conjugated anti-VHH antibody. Probing with an anti-His antibody served as a control (HIS). A Coomassie-stained SDS-PAGE gel of recombinant PfPIMMS43 prior to blotting was used as a loading control (COO). The full and unedited images of western blots are shown.

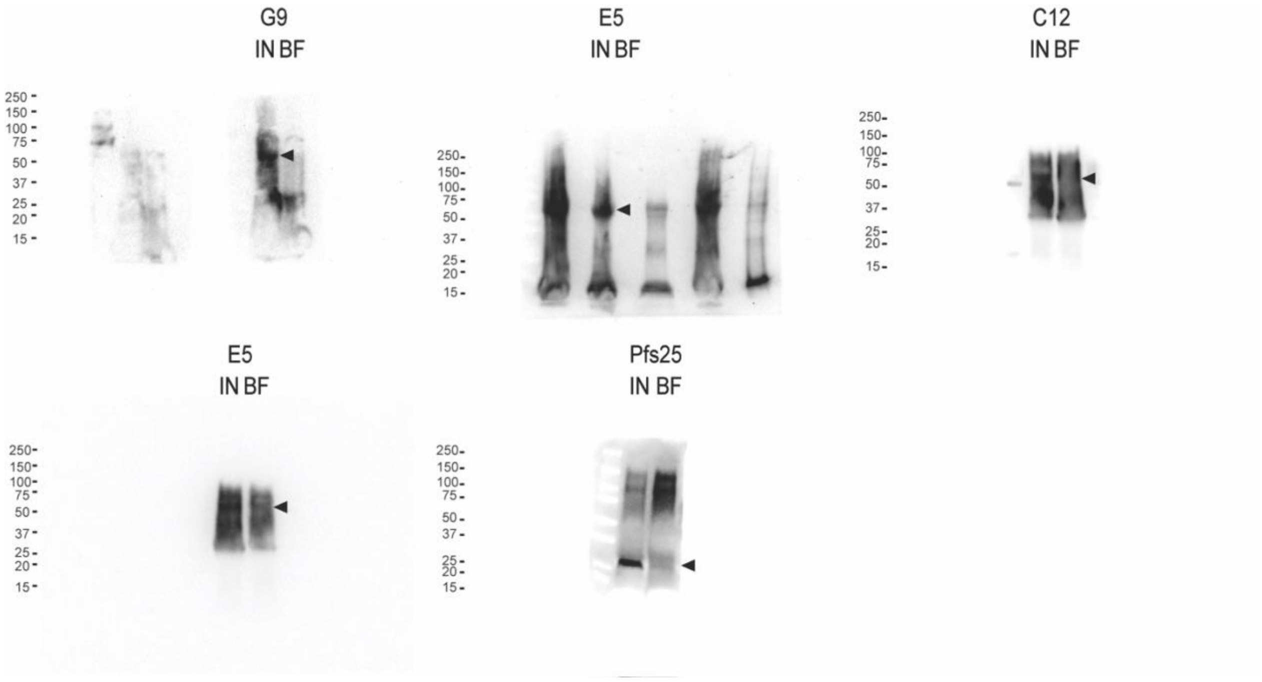

Figure S2. Nanobody detection of endogenous PfPIMMS43 in *P. falciparum* NF54.
Reduced cell lysates from *P. falciparum*-infected midguts, collected 18 hours post-blood meal, were probed with the four highest affinity nanobodies (G9, E5, C12, and E2) and binding was detected using an anti-MYC antibody. Detection of Pfs25 with a specific antibody is used as a control. The full and unedited western blots are shown.

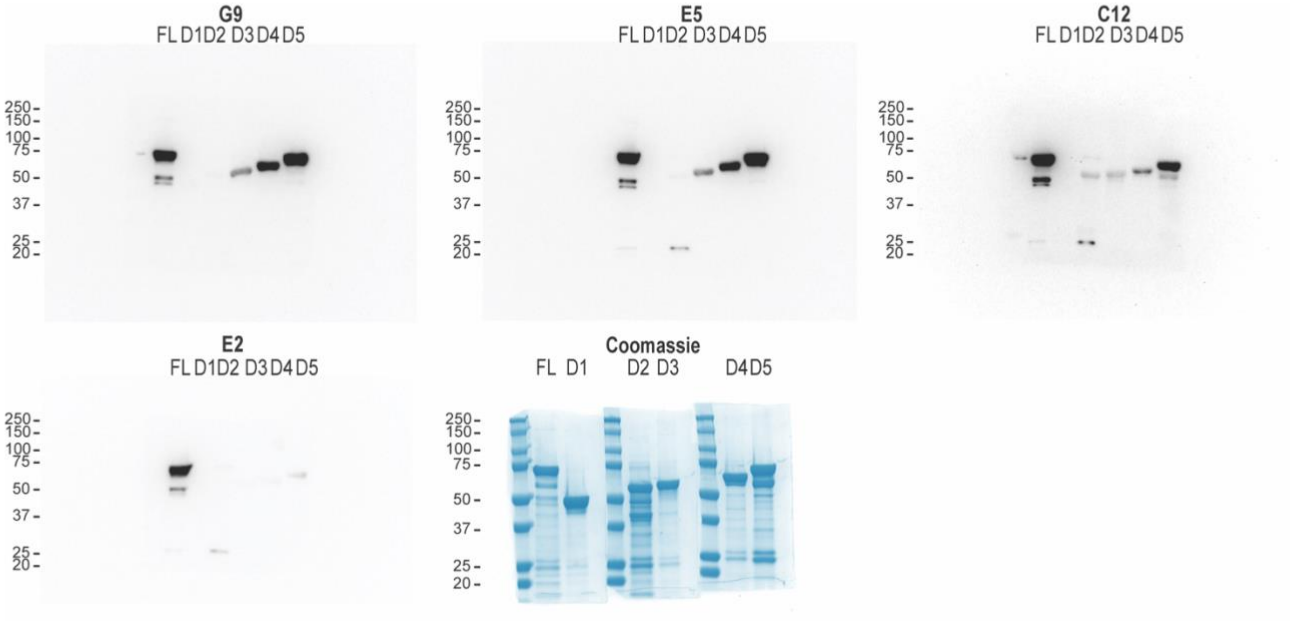

Figure S3. PfPIMMS43 epitope mapping of VHHs.
Western blot detection of recombinant Thioredoxin-His-tagged PfPIMMS43 FL and its truncated variants D1-D5 using G9, E5, C12, and E2 nanobodies. Each nanobody was tested individually, and binding was detected using an HRP-conjugated anti-VHH antibody. A Coomassie-stained SDS-PAGE gel of the recombinant PfPIMMS43 proteins, served as a loading control. The uncropped and unedited images of western blots are shown.

Supplementary Tables

Table S1. Transmission blocking effect of PfPIMMS43 nanobodies on *P. falciparum* NF54 using SMFAs in *An. coluzzii*.

VHH (Replicate)	[VHH] (µg/mL)	Midgut numbers	Prevalence (%)	Prevalence P-value	Mean (TRA%)	Median	Oocyst range	Intensity P-value
C12 (R1)	PBS	47	36		1	0	0-7	
	25	52	37	>0.9999	0.7 (30↓)	0	0-9	0.8526
	50	42	5	0.0003	0.1 (90↓)	0	0-2	0.0003
	100	49	0	<0.0001	0 (100↓)	0	0	<0.0001
C12 (R2)	PBS	49	41		1.5	0	0-12	
	25	50	34	0.5368	1.1 (27↓)	0	0-10	0.2958
	50	50	20	0.0297	0.6 (60↓)	0	0-8	0.0173
	100	49	14	0.0061	0.3 (80↓)	0	0-5	0.0014
C12 (R3)	PBS	48	44		4.3	0	0-30	
	25	47	36	0.5315	2.9 (33↓)	0	0-28	0.3687
	50	49	51	0.5439	3.3 (23↓)	1	0-18	0.8626
	100	44	14	0.0025	0.6 (86↓)	0	0-7	0.0008
C12 (All)	PBS	144	40		2.3	0	0-30	
	25	149	36	0.47	1.5 (30↓)	0	0-28	0.2012
	50	141	26	0.0124	1.4 (58↓)	0	0-18	0.011
	100	142	9	<0.0001	0.3 (89↓)	0	0-7	<0.0001
E2 (R1)	PBS	51	51		2.5	1	0-23	
	25	49	16	<0.0001	1.1 (56↓)	0	0-20	0.0005
	50	27	15	0.0029	0.7 (72↓)	0	0-7	0.0053
	100	16	0	<0.0001	0 (100↓)	0	0	0.0004
E2 (R2)	PBS	52	33		1.1	0	0-11	
	25	43	14	0.053	0.3 (73↓)	0	0-5	0.0297
	50	35	14	0.0776	0.5 (55↓)	0	0-6	0.0664
	100	25	28	0.7952	0.5 (55↓)	0	0-3	0.5295
E2 (R3)	PBS	49	71		26.6	22	0-136	
	25	51	55	0.1008	8.4 (68↓)	1	0-62	0.0009
	50	37	24	<0.0001	2.4 (91↓)	0	0-32	<0.0001
	100	19	16	<0.0001	1.8 (93↓)	0	0-24	<0.0001
E2 (All)	PBS	152	51		9.8	1	0-136	
	25	143	29	<0.0001	3.5 (66↓)	1	0-62	<0.0001
	50	99	18	<0.0001	1.3 (73↓)	0	0-32	<0.0001
	100	60	17	<0.0001	0.8 (83↓)	0	0-24	<0.0001
E5 (R1)	PBS	46	43		4.1	0	0-83	
	25	48	56	0.3022	8.0 (49↑)	1.5	0-50	0.0409
	50	61	38	0.5575	3.5 (15↓)	0	0-41	0.773
	100	43	5	<0.0001	0.1 (98↓)	0	0-3	<0.0001
E5 (R2)	PBS	49	39		1	0	0-12	
	25	50	18	0.0266	1.1 (10↑)	0	0-16	0.0508
	50	50	14	0.0063	0.4 (60↓)	0	0-10	0.005
	100	36	0	<0.0001	0 (100↓)	0	0	<0.0001

E5 (R3)	PBS	49	53		3.9	1	0-26	
	25	52	35	0.1105	1.6 (59↓)	0	0-18	0.0352
	50	49	31	0.0637	1.1 (72↓)	0	0-9	0.017
	100	16	0	0.0002	0 (100↓)	0	0	0.0004
E5 (All)	PBS	144	44		3	0	0-83	
	25	144	35	0.119	3.5 (0)	0	0-50	0.3585
	50	145	28	0.0033	1.9 (49↓)	0	0-41	0.0054
	100	95	2	<0.0001	0 (99↓)	0	0-3	<0.0001
G9 (R1)	PBS	48	67		11.5	7	0-64	
	25	48	31	0.001	3.9 (66↓)	0	0-37	0.0003
	50	48	33	0.002	2.1 (82↓)	0	0-21	<0.0001
	100	47	15	<0.0001	0.4 (97↓)	0	0-8	<0.0001
G9 (R2)	PBS	49	27		1	0	0-12	
	25	50	16	0.227	0.4 (60↓)	0	0-8	0.1663
	50	48	8	0.0307	0.3 (70↓)	0	0-9	0.0233
	100	51	4	0.0017	0.1 (90↓)	0	0-2	0.0008
G9 (R3)	PBS	51	61		5.2	1	0-24	
	25	49	33	0.0055	2.7 (48↓)	0	0-36	0.005
	50	51	43	0.1124	2.2 (58↓)	0	0-26	0.0231
	100	50	30	0.0027	1.5 (71↓)	0	0-21	0.0007
G9 (All)	PBS	148	51		5.8	1	0-64	
	25	147	27	<0.0001	2.3 (58↓)	0	0-37	<0.0001
	50	147	29	<0.0001	1.6 (70↓)	0	0-26	<0.0001
	100	148	16	<0.0001	0.7 (86↓)	0	0-21	<0.0001

Oocyst data collected 8-10 days post mosquito blood feeding on PBS- or nanobody-spiked *P. falciparum* NF54 gametocytes. P values for prevalence were calculated using the Fisher's exact test and for infection intensities using the Mann-Whitney U test. Brackets show the percent transmission reduction activity (TRA) of each nanobody compared to respective PBS controls.

Table S2. Parasitological data of *P. falciparum* gametocyte carriers used in DMFA's

Replicate	ABS (per 200 WBC)	Gametocyte (per µL blood)
1	7	48
2	8	32
3	15	48

The calculation of gametocytes per µL of blood assumes a white blood cell (WBC) count of 8000 per µL of blood. ABS shows the density of asexual blood stages (trophozoites) per 200 WBC.

Table S3. Transmission blocking effect of PfPIMMS43 nanobodies on *P. falciparum* natural isolates using DMFAs in *An. gambiae*.

VHH (Replicate)	[VHH] (µg/mL)	Midgut numbers	Prevalence (%)	Prevalence P-value	Mean (TRA%)	Median	Oocyst range	Intensity P-value
E5 (R1)	PBS	104	60		2.1	1	0-15	

	25	15	20	0.0051	0.9 (55)	0	0-10	0.0108
	50	30	17	<0.0001	0.6 (73)	0	0-6	0.0001
	100	41	24	0.0002	0.4 (79)	0	0-3	<0.0001
E5 (R2)	PBS	117	55		1.2	1	0-9	
	25	24	17	0.0007	0.3 (79)	0	0-3	0.0007
	50	30	10	<0.0001	0.2 (86)	0	0-2	<0.0001
	100	41	24	0.001	0.3 (77)	0	0-2	0.0002
E5 (R3)	PBS	113	52		3.6	3	0-18	
	25	39	23	0.0016	1.3 (64)	0	0-13	0.001
	50	35	14	<0.0001	1.1 (70)	0	0-10	0.0003
	100	39	15	<0.0001	0.7 (80)	0	0-8	<0.0001
E5 (All)	PBS	334	55		2.3	1	0-18	
	25	78	21	<0.0001	0.9 (66)	0	0-13	<0.0001
	50	95	14	<0.0001	0.6 (76)	0	0-10	<0.0001
	100	121	21	<0.0001	0.5 (79)	0	0-8	<0.0001
G9 (R1)	PBS	104	60		2.1	1	0-15	
	25	29	48	0.2956	1.3 (39)	0	0-9	0.1507
	50	33	27	0.0014	1 (51)	0	0-10	0.0043
	100	35	3	<0.0001	0 (99)	0	0-1	<0.0001
G9 (R2)	PBS	117	55		1.2	1	0-9	
	25	29	21	0.0015	0.2 (79)	0	0-2	0.0005
	50	33	12	<0.0001	0.2 (87)	0	0-2	<0.0001
	100	35	0	<0.0001	0 (100)	0	0-0	<0.0001
G9 (R3)	PBS	113	52		3.6	3	0-18	
	25	32	38	0.164	1.8 (51)	0	0-9	0.0361
	50	33	15	0.0001	1.2 (66)	0	0-10	0.001
	100	35	6	<0.0001	0.1 (98)	0	0-1	<0.0001
G9 (All)	PBS	334	55		2.3	1	0-18	
	25	90	36	0.0009	1.1 (56)	0	0-9	0.0003
	50	99	18	<0.0001	0.8 (68)	0	0-10	<0.0001
	100	105	3	<0.0001	0 (99)	0	0-1	<0.0001

Oocyst data collected 8-10 days post mosquito blood feeding on PBS- or nanobody-spiked *P. falciparum* gametocytes sampled from infected children. P values for prevalence were calculated using the Fisher's exact test and for infection intensities using the Mann-Whitney U test.
