# Supplementary material for: Nanobody-mediated targeting of Plasmodium falciparum PfPIMMS43 can block malaria transmission in mosquitoes
Source: Commun Biol. 2025 Apr 30;8:683. doi: 10.1038/s42003-025-08033-8 (PMC12041390; doi:10.1038/s42003-025-08033-8)
Supplement: Supplementary file 2 — Description of Additional Supplementary Materials [file 42003_2025_8033_MOESM2_ESM.pdf]

Description of Additional Supplementary Files

File name: Supplementary Data 1

Description: Raw data from infections with laboratory NF54 (spreadsheet 1) and natural P. falciparum isolates

File name: Supplementary Data 2

Description: Mass spectrometry analysis of peptides obtained after tryptic digest of VHH-PfPIMMS43 complex.
