# Supplementary material for: Nanobody-mediated targeting of Plasmodium falciparum PfPIMMS43 can block malaria transmission in mosquitoes
Source: Commun Biol. 2025 Apr 30;8:683. doi: 10.1038/s42003-025-08033-8 (PMC12041390; doi:10.1038/s42003-025-08033-8)
Supplement: Supplementary file 5 — Reporting Summary [file 42003_2025_8033_MOESM5_ESM.pdf]

nature portfolio

Corresponding author(s): Dina Vlachou

Last updated by author(s): 3/19/2025

Reporting Summary

Nature Portfolio wishes to improve the reproducibility of the work that we publish. This form provides structure for consistency and transparency in reporting. For further information on Nature Portfolio policies, see our Editorial Policies and the Editorial Policy Checklist.

Statistics

For all statistical analyses, confirm that the following items are present in the figure legend, table legend, main text, or Methods section.

n/a	Confirmed	
☐	☒	The exact sample size (*n*) for each experimental group/condition, given as a discrete number and unit of measurement
☐	☒	A statement on whether measurements were taken from distinct samples or whether the same sample was measured repeatedly
☐	☒	The statistical test(s) used AND whether they are one- or two-sided<br>*Only common tests should be described solely by name; describe more complex techniques in the Methods section.*
☐	☒	A description of all covariates tested
☐	☒	A description of any assumptions or corrections, such as tests of normality and adjustment for multiple comparisons
☐	☒	A full description of the statistical parameters including central tendency (e.g. means) or other basic estimates (e.g. regression coefficient) AND variation (e.g. standard deviation) or associated estimates of uncertainty (e.g. confidence intervals)
☐	☒	For null hypothesis testing, the test statistic (e.g. *F*, *t*, *r*) with confidence intervals, effect sizes, degrees of freedom and *P* value noted<br>*Give P values as exact values whenever suitable.*
☒	☐	For Bayesian analysis, information on the choice of priors and Markov chain Monte Carlo settings
☒	☐	For hierarchical and complex designs, identification of the appropriate level for tests and full reporting of outcomes
☒	☐	Estimates of effect sizes (e.g. Cohen's *d*, Pearson's *r*), indicating how they were calculated

Our web collection on statistics for biologists contains articles on many of the points above.

Software and code

Policy information about availability of computer code

Data collection	N/A
Data analysis	N/A

For manuscripts utilizing custom algorithms or software that are central to the research but not yet described in published literature, software must be made available to editors and reviewers. We strongly encourage code deposition in a community repository (e.g. GitHub). See the Nature Portfolio guidelines for submitting code & software for further information.

Data

Policy information about availability of data

All manuscripts must include a data availability statement. This statement should provide the following information, where applicable:

- Accession codes, unique identifiers, or web links for publicly available datasets
- A description of any restrictions on data availability
- For clinical datasets or third party data, please ensure that the statement adheres to our policy

All data supporting the findings of this study are available within the paper and its Supplementary Information

Research involving human participants, their data, or biological material

Policy information about studies with human participants or human data. See also policy information about sex, gender (identity/presentation), and sexual orientation and race, ethnicity and racism.

Reporting on sex and gender	Human participants were chosen using fully inclusive protocols. Sex and gender were not taken into account during the study design, were not analyzed separately, and were neither self-reported nor assigned by the researchers.
Reporting on race, ethnicity, or other socially relevant groupings	Human participants were selected using fully inclusive protocols. Factors such as race, ethnicity, and other socially relevant categories were not considered in the study design, were not analyzed separately, and were neither self-reported nor assigned by the researchers.
Population characteristics	Participants were children aged 6 to 14. No other demographic characteristics were considered in the study design or data analysis.
Recruitment	Participants were recruited from primary schools in Wami Mkoko and Miono villages during the school term. Recruitment was carried out without considering sex, gender, sexual orientation, race, or ethnicity, ensuring an inclusive approach.
Ethics oversight	The study protocol was reviewed and approved by the IHI Institutional Biosafety Committee (IBC) and the National Institute for Medical Research, Tanzania. Written informed consent was obtained from parents or guardians, while oral assent was secured from the children. The research complied with ethical guidelines and regulations, including the Declaration of Helsinki, ensuring that participants' rights to withdraw from the study and to maintain their privacy were respected throughout the research process.

Note that full information on the approval of the study protocol must also be provided in the manuscript.

Field-specific reporting

Please select the one below that is the best fit for your research. If you are not sure, read the appropriate sections before making your selection.

☒ Life sciences ☐ Behavioural & social sciences ☐ Ecological, evolutionary & environmental sciences

For a reference copy of the document with all sections, see nature.com/documents/nr-reporting-summary-flat.pdf

Life sciences study design

All studies must disclose on these points even when the disclosure is negative.

Sample size	Sample sizes were determined based on the experimental requirements. Each DMFA included 3 cups, each containing 60 female mosquitoes, for 3 different concentrations of nanobody treated mosquitoes, along with a separate cup for non treated mosquitoes. Gametocyte carriers were selected from a pool of 40–50 children, focusing on individuals with more than 10 gametocytes per microliter, specifically at stage V development, with an equal distribution of male and female gametocytes.
Data exclusions	N/A
Replication	Experimental were performed in triplicate, with survival and reproductive fitness assays conducted across multiple replicate groups and parasite infection experiments repeated across different infection replicates.
Randomization	N/A
Blinding	N/A

Reporting for specific materials, systems and methods

We require information from authors about some types of materials, experimental systems and methods used in many studies. Here, indicate whether each material, system or method listed is relevant to your study. If you are not sure if a list item applies to your research, read the appropriate section before selecting a response.

Materials & experimental systems

n/a	Involved in the study
☐	☒ Antibodies
☒	☐ Eukaryotic cell lines
☒	☐ Palaeontology and archaeology
☐	☒ Animals and other organisms
☐	☒ Clinical data
☒	☐ Dual use research of concern
☒	☐ Plants

Methods

n/a	Involved in the study
☒	☐ ChIP-seq
☒	☐ Flow cytometry
☒	☐ MRI-based neuroimaging

Antibodies

Antibodies used	c-Myc (D84C12) Rabbit mAb, Cell Signaling Technologies, #5605<br>Rabbit Anti-Camelid VHH Antibody, mAb, Genescript, A01860<br>4D7 mouse monoclonal α-Pfs25, produced in house from hybridoma<br>Anti-Rabbit IgG (H+L), HRP Conjugate, Promega, W4011<br>Promega's Anti-Mouse IgG (H+L), HRP Conjugate, Promega, W4021
Validation	N/A

Animals and other research organisms

Policy information about studies involving animals; ARRIVE guidelines recommended for reporting animal research, and Sex and Gender in Research

Laboratory animals	No animals were used in-house for this study. The nanobodies were derived from the immunization of two llamas (Lama glama), conducted by Eurogentec. The immunization and lymphocyte harvesting procedures adhered to Eurogentec's approved protocols and ethical guidelines for animal welfare.
Wild animals	N/A
Reporting on sex	Sex was not considered relevant to this protocol, and the company has not provided information regarding the sex of the two animals.
Field-collected samples	N/A
Ethics oversight	For more details on Eurogentec's animal facilities and ethical standards, please refer to their website: https://www.eurogentec.com/en/animal-facilities.

Note that full information on the approval of the study protocol must also be provided in the manuscript.

Clinical data

Policy information about clinical studies

All manuscripts should comply with the ICMJE guidelines for publication of clinical research and a completed CONSORT checklist must be included with all submissions.

Clinical trial registration	N/A
Study protocol	The study involved parasitological surveys of children in Tanzanian villages to assess malaria prevalence and collect blood samples for mosquito infection experiments.
Data collection	Parasitological data were collected through Rapid Diagnostic Testing (RDT), thick blood smear microscopy and gametocyte density assessments.
Outcomes	Effectiveness of treated mosquitoes with nanobodies in inhibiting parasite transmission

Plants

Seed stocks	N/A
Novel plant genotypes	N/A
Authentication	N/A
